# IRF1-mediated immune cell infiltration is associated with metastasis in colon adenocarcinoma

**DOI:** 10.1097/MD.0000000000022170

**Published:** 2020-09-11

**Authors:** Yao-jian Shao, Jun-jie Ni, Shen-yu Wei, Xiong-peng Weng, Meng-die Shen, Yi-xin Jia, Li-na Meng

**Affiliations:** a First College of Clinical Medical, Zhejiang Chinese Medical University, Hangzhou; b Second College of Clinical Medical, Wenzhou Medical University, Wenzhou; c Department of Gastroenterology, the First Affiliated Hospital of Zhejiang Chinese Medical University; d Key Laboratory of Digestive Pathophysiology of Zhejiang Province, Hangzhou, Zhejiang, PR China.

**Keywords:** colon adenocarcinoma, immune cell infiltration, IRF1, metastasis

## Abstract

**Background:**

Evidence suggests that metastasis is chiefly responsible for the poor prognosis of colon adenocarcinoma (COAD). The tumor microenvironment plays a vital role in regulating this biological process. However, the mechanisms involved remain unclear. The aim of this study was to identify crucial metastasis-related biomarkers in the tumor microenvironment and investigate its association with tumor-infiltrating immune cells.

**Methods:**

We obtained gene expression profiles and clinical information from The Cancer Genome Atlas database. According to the “Estimation of STromal and Immune cells in MAlignant Tumor tissue using Expression data” algorithm, each sample generated the immune and stromal scores. Following correlation analysis, the metastasis-related gene was identified in The Cancer Genome Atlas database and validated in the GSE40967 dataset from Gene Expression Omnibus. The correlation between metastasis-related gene and infiltrating immune cells was assessed using the Tumor IMmune Estimation Resource database.

**Results:**

The analysis included 332 patients; the metastatic COAD samples showed a low immune score. Correlation analysis results showed that interferon regulatory factor 1 (IRF1) was associated with tumor stage, lymph node metastasis, and distant metastasis. Furthermore, significant associations between IRF1 and CD8+ T cells, T cell (general), dendritic cells, T-helper 1 cells, and T cell exhaustion were demonstrated by Spearmans correlation coefficients and *P* values.

**Conclusions:**

The present findings suggest that IRF1 is associated with metastasis and the degree of immune infiltration of CD8+ T cells (general), dendritic cells, T-helper 1 cells, and T cell exhaustion in COAD. These results may provide information for immunotherapy in colon cancer.

## 1 Introduction

Colorectal cancer is a common malignancy of the digestive system, with approximately 147,950 new cases and 53,200 deaths reported in United States of America in 2020.^[1]^ Colon adenocarcinoma (COAD) is the predominant pathological classification of colorectal cancer, accounting for >90% of cases.^[2]^ The stage of patients at diagnosis is an important predictor of prognosis. The 5-year survival rate ranges from 91% (for patients diagnosed with stage I disease) to 12% (for those diagnosed with stage IV disease).^[3]^ The limited effectiveness of treatments in patients with metastatic is responsible for the high mortality rates observed. Accordingly, it is important to investigate the etiopathogenesis of metastasis and develop effective therapies for COAD with metastasis.

Accumulating evidence indicates that the tumor microenvironment (TME) regulates numerous facets of tumor progression, including cell proliferation and metastasis.^[4,5]^ The TME is a dynamic system composed of immune cells, stromal cells, cytokines, and extracellular matrix. Among them, immune and stromal cells play indispensable roles as primary producers of cytokines and chemokines.^[6]^ Different degrees of infiltration of immune cells can alter tumor behavior. The antigen-presenting cells with PD-L1 positivity are able to promote the metastasis of tumor cells by impairing the cytotoxicity PD-L1 against CD8+ T cells.^[7,8]^ T cell chemokines (such as C-C motif chemokine ligand 3 [CCL3], C-X-C motif chemokine ligand 9 [CXCL9], and CXCL10) and proinflammatory cytokines (such as interleukin 12 [IL12], tumor necrosis factor alpha [TNFα], and granulocyte-macrophage colony stimulating factor [GM-CSF]) secreted by CD8+T cells are capable of killing tumor cells.^[9,10]^ CD8+ T cells can be activated by neutrophils in the “N1” polarization state to enhance the anti-tumor effect. Nevertheless, the anti-tumor immunity is impaired by neutrophils in the “N2” polarization state, such as the action of pathological Notch signal secretion and regulatory T cell recruitment.^[10–12]^ Fibroblasts are the main representative components of stromal cells. Activin A secreted by fibroblasts is a powerful pro-metastatic cytokine, which induces the migration of epithelial cells.^[13]^ Fibroblast-derived CXCL12 alters PI3K/AKT signaling transduction to influence the aggressive spread of COAD.^[14]^ Recently, immunotherapeutic strategies, such as programmed cell death-1/programmed cell death ligand-1 (PD-1/PD-L1) have been developed and were shown to be effective against non-small cell lung cancer,^[15]^ melanoma,^[16]^ kidney cancer,^[17]^ and microsatellite instability colorectal cancer.^[18]^ However, weak responses to treatment with PD-1/PD-L1 checkpoint inhibitors persist in other subtypes of colorectal cancer.

The Estimation of STromal and Immune cells in Malignant Tumor tissues using Expression data (ESTIMATE) algorithm can help to reveal the infiltration degree of immune and stromal cells in the TME.^[19]^ The immune and stromal score of each sample represents the infiltration degree of immune and stromal cells. The ESTIMATE algorithm has been employed in glioblastoma,^[20]^ bladder cancer,^[21]^ and renal clear cell carcinoma.^[22]^

In this study, we obtained 332 complete gene expression profiles of COAD from The Cancer Genome Atlas (TCGA) database and calculated the immune and stromal scores based on the ESTIMATE algorithm. The tumor metastasis-related genes (MRGs) were extracted following verification in the Gene Expression Omnibus (GEO) database. Based on 2 dataset, interferon regulatory factor 1 (IRF1) was differentially expressed in different stages of COAD, and was considered the MRG in COAD. IRF1 is a member of the interferon regulatory factor family; the protein encoded by this gene is considered a tumor suppressor by stimulating the immune response against tumors. Previous research studies have reported that IRF1 can affect the maturation and activity of natural killer cells, development of T helper 1 (Th1) cells, and maturation of CD8+ T-cells. Therefore, the role of IRF1 in the COAD immune microenvironment was further explored in the Tumor IMmune Estimation Resource (TIMER) site.

## 2 Materials and methods

### 2.1 Database

The dataset including the gene expression profile (RNA, Seq V3) and corresponding clinical information of 385 patients with COAD was downloaded from TCGA (https://gdc.nci.nih.gov/). After excluding patients with indistinct clinical information, we included 332 samples in the analysis. The ESTIMATE algorithm was applied to calculate the immune/stromal scores of each sample. We analyzed the distribution of immune/stromal scores from 3 different groups: tumor stage, lymph node metastasis (N), and distant metastasis (M) using the R package. Tumors were classified into stages I, II, III, and IV; lymph node metastases were divided into N0 and N1–2 groups; and distant metastases were divided into M0 and M1 groups.

### 2.2 Identification of differentially expressed genes (DEGs)

According to the immune/stromal scores of the 332 samples, we classified them into high- and low-score groups. The R package Limma was utilized to screen DEGs. Absolute values of log fold-change (logFC) ≥ 1 and false discovery rate <.05 were set as thresholds to filter DEGs.

### 2.3 Construction of a protein-protein interaction (PPI) network

We collected information on proteins encoded by DEGs and constructed a PPI network with the highest confidence (.95) interaction score in the STRING database (http://string-db.org). Subsequently, the PPI network was imported into the Cytoscape software for reestablishment. The MCODE plugin of Cytoscape was applied to identify the significant modules, which were the densely connected regions of the PPI network.

### 2.4 Enrichment analyses of function and pathway

Functional and pathway analyses of DEGs contained in significant modules were performed using the R package clusterProfiler. The Gene Ontology (GO) includes 3 sub ontologies, namely biological process (BP), cellular component (CC), and molecular function (MF). The adjusted *P* value <.05 was set as the cutoff value for GO term and Kyoto Encyclopedia of Genes and Genomes (KEGG) pathway analysis. The top 10 terms of each sub ontology are shown in the bubble diagram.

### 2.5 Metastasis correlation analysis and verification

Metastasis correlation analysis was performed using the R package beeswarm and a boxplot was used to exhibit the expression of DEGs in the tumor stage group, lymph node metastasis group and distant metastasis group. *P* values < .05 denoted statistical significance. We adopted the GSE40967 dataset from the GEO database to verify the characteristics of DEG expression using the same approach. The GSE40967 dataset contains the gene expression profiles and clinical information of 1048 patients with COAD based on the GPL570 platforms (Affymetrix Human Genome U133 Plus 2.0 Array). Finally, only 560 samples with complete TNM stage clinical information were selected for the correlation analysis. The DEGs with significant differences in TCGA database and GEO database were considered MRGs. The prognostic value of MRGs was explored using the Gene Expression Profiling Interactive Analysis (GEPIA; http://gepia.cancer-pku.cn/) online tool, and was shown via a Kaplan–Meier survival curve.

### 2.6 Immune infiltration

The TIMER database (https://cistrome.shinyapps.io/timer/) was utilized to assess the correlation of the expression of MRGs with different types of immune infiltrates. In the gene module, we analyzed the correlation of MRG expression with tumor-infiltrating immune cells of COAD. In addition, we investigated the correlation between MRG expression and gene markers of diverse immune cells, which were referenced in previous studies via a correlation module.^[23]^ We classified infiltrating immune cells in detail according to their different functions. The macrophages were subdivided into tumor-associated, M1, and M2 macrophages. T cells were subdivided into CD8+ T, T (general), Th1, Th2, follicular T helper, Th17, regulatory T cells, and T cell exhaustion. The scatter plots together with Spearmans correlation coefficients and *P* values were generated in both modules to show the associations. The expression of MRGs and levels of gene markers in scatter plots were detected using log2 RSEM. The absolute value of Spearmans correlation coefficient determined the strength of the correlation based on the following guideline (weak: <.40; moderate: .40–.59; strong: >.60). *P* values <.05 denoted statistical significance.

### 2.7 Ethics statement

The datasets in this study are achieved form publicly available database (TCGA and GEO). Ethical approval and informed consent were thus not required.

## 3 Results

### 3.1 Immune cell infiltrations are correlated with the clinical characteristics of COAD

**Figure 1:**
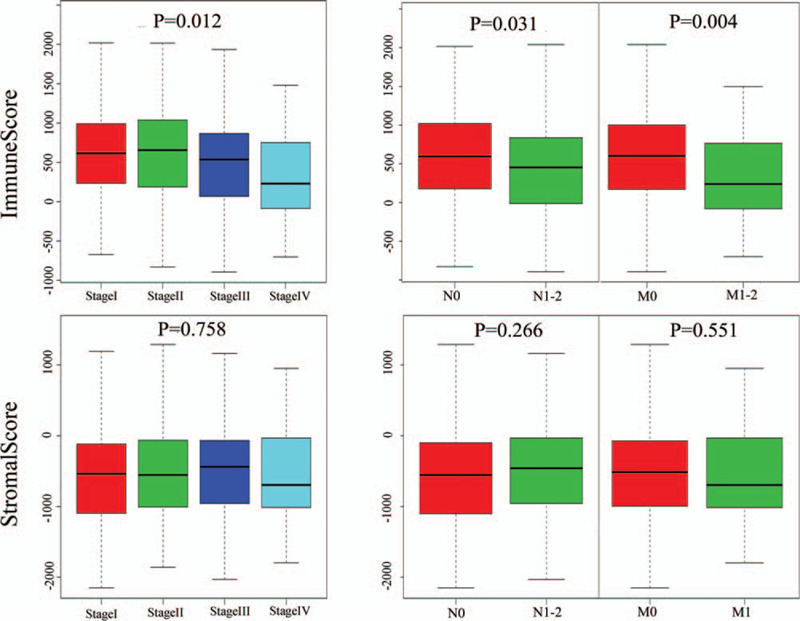
Clinical correlation analysis of the immune score and stromal score in COAD. The immune score showed significant correlations with stage (*P* = .012), lymphatic metastasis (*P* = .031), and distant metastasis (*P* = .004); however, there was no statistically significant difference found in the stromal scores. COAD = colon adenocarcinoma.

A total of 332 COAD samples with specific gene expression and clinical information were included in this study. The stages of disease for these patients were: stage I (n = 58, 17.5%), stage II (n = 135, 40.6%), stage III (n = 86, 25.9%), and stage IV (n = 53, 16.0%). The details of TNM staging and other characteristics are listed in Table 1. We calculated the immune and stromal scores of 332 samples by employing the ESTIMATE algorithm. The distribution of the immune and stromal scores was between −893.55 to 2417.00 and −2151.29 to 1696.53, respectively. To explore the value of infiltrating immune and stromal cells in COAD, we assessed the association between immune/stromal scores and tumor stage, lymph node metastasis, and distant metastasis using the R package. As shown in Figure 1, there was no statistically significant difference found in the stromal scores, whereas the immune score was markedly correlated with stage (*P* = .012), lymph node metastasis (*P* = .031) and distant metastasis (*P* = .004). The distribution of the immune score was as follows: stages I and II > stage III > stage IV. The preliminary investigation revealed that the patients with lymph node metastasis and distant metastasis generated lower immune scores. Subsequently, we selected the immune score for the following studies.

**Table 1:**
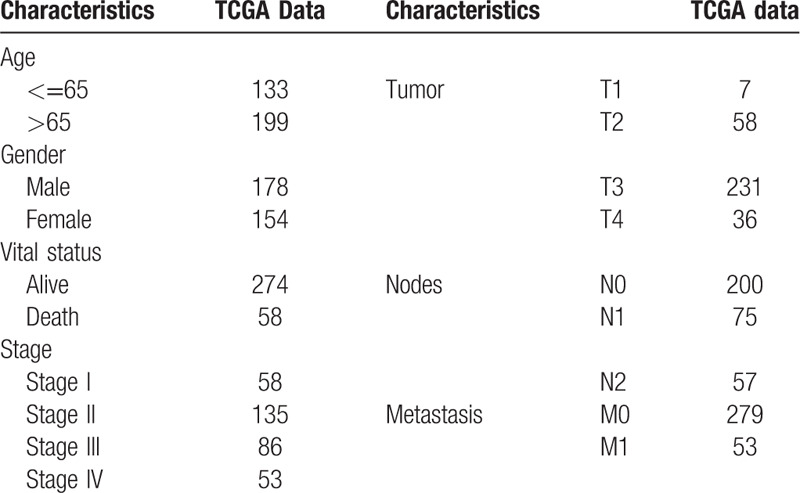
Characteristics of COAD patients in TCGA database.

### 3.2 DEG screening based on the immune score

**Figure 2:**
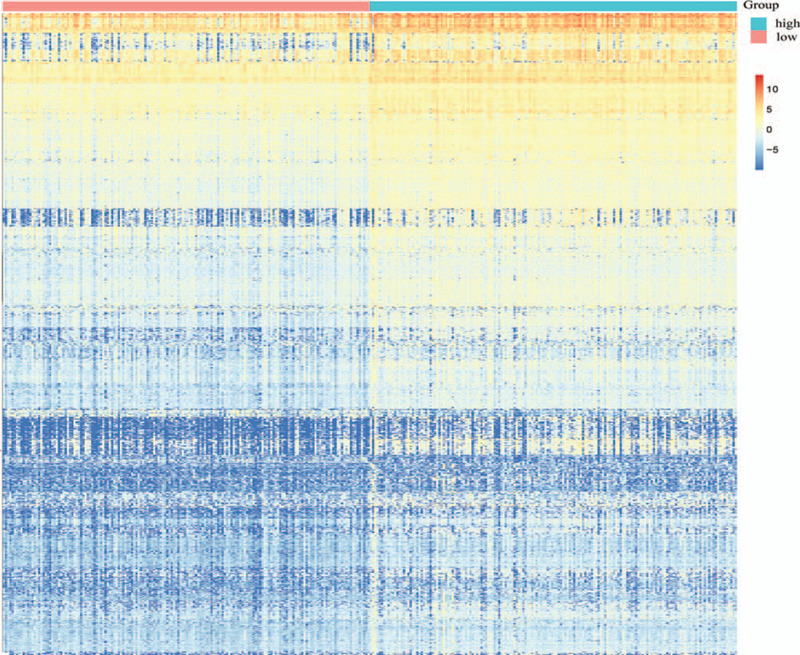
Heatmap of the immune scores of 1,655 DEGs (1608 upregulated DEGs and 47 downregulated DEGs). DEG = differentially-expressed gene.

To determine the potential association of immune cell infiltration with gene expression profiles, all COAD samples were divided into 2 different groups by setting the median of the immune score as the threshold value. The differential expression analysis between high and low immune scores was performed using the R package Limma. According to the cutoff criteria (false discovery rate <.05 and |logFC| ≥ 1.0), we obtained 1,655 DEGs (1,608 upregulated DEGs and 47 downregulated DEGs). Figure 2 illustrates the heatmap of DEGs.

### 3.3 Three significant modules from the PPI network

**Figure 3:**
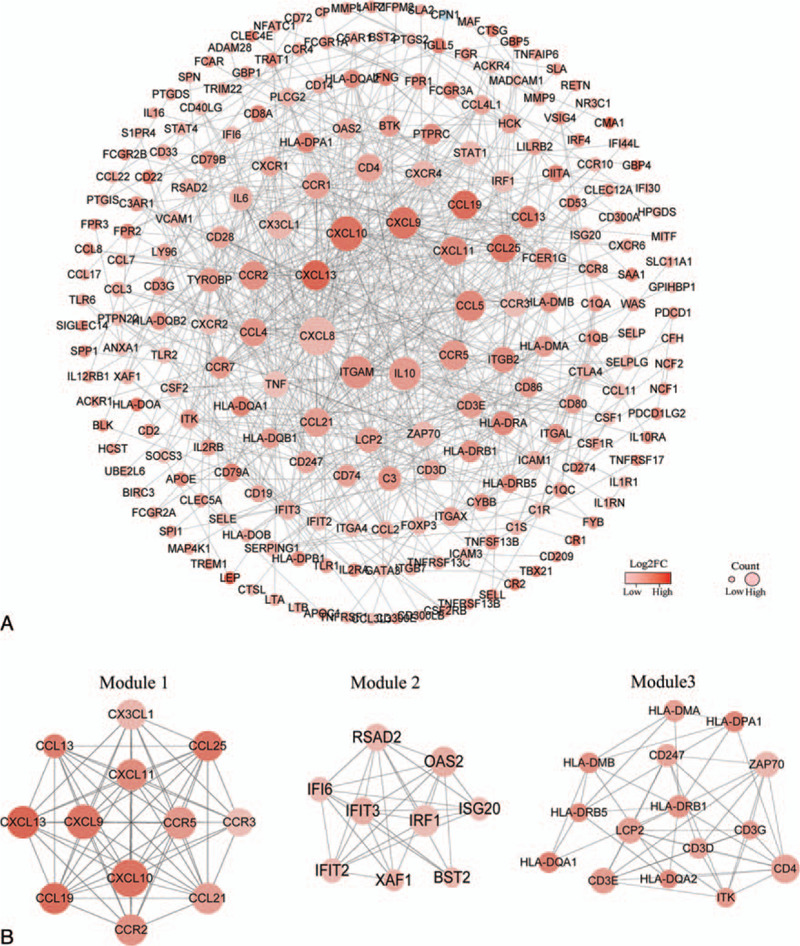
(A) After removing outlying genes without interaction, the PPI network consisted of 222 DEGs. Node color indicates the log2FC of gene expression and node size increased based on the count of interacting proteins. (B) Top 3 significant modules of the PPI network. Module 1 consisted of 12 nodes and 63 edges. Module 2 consisted of 9 nodes and 29 edges. Module 3 consisted of 15 nodes and 46 edges. DEG = differentially-expressed gene, FC = fold change, PPI = protein-protein interaction.

The PPI network of DEGs was constructed using the STRING database and Cytoscape software (Version 3.7.1). After removing the outlying genes without interaction, the network contained 222 nodes and 674 edges. Node color indicated the log2FC of gene expression and the node size increased based on the count of interacting proteins (Fig. 3A). Subsequently, the MCODE plugin of Cytoscape was utilized to identify significant modules of the PPI network; the top 3 modules were identified and termed Modules 1, 2, and 3 for convenience. Module 1 consisted of 12 nodes and 63 edges, including CXCL9, CXCL13, CXCL11, CXCL10, CX3CL1, CCR5, CCR3, CCR2, CCL25, CCL21, CCL19, and CCL13. Module 2 contained 29 edges involving 9 nodes: bone marrow stromal cell antigen 2 (BST2), interferon alpha inducible protein 6 (IFI6), interferon induced protein with tetratricopeptide repeats 2 (IFIT2), IFIT3, IRF1, interferon stimulated exonuclease gene 20 (ISG20), 2’-5’-oligoadenylate synthetase 2 (OAS2), radical S-adenosyl methionine domain containing 2 (RSAD2), and XIAP associated factor 1 (XAF1). In Module 3, 46 edges were formed in the network involving 15 nodes, namely CD247, CD3D, CD3E, CD3G, CD4, HLA-DMA, HLA-DMB, HLA-DPA1, HLA-DQA1, HLA-DQA2, HLA-DRB1, HLA-DRB5, IL2 inducible T cell kinase (ITK), lymphocyte cytosolic protein 2 (LCP2), and zeta chain of T cell receptor associated protein kinase 70 (ZAP70). The DEGs contained in the 3 modules were all upregulated in the high immune score group (Fig. 3B).

### 3.4 GO term and KEGG pathway enrichment analyses

**Figure 4:**
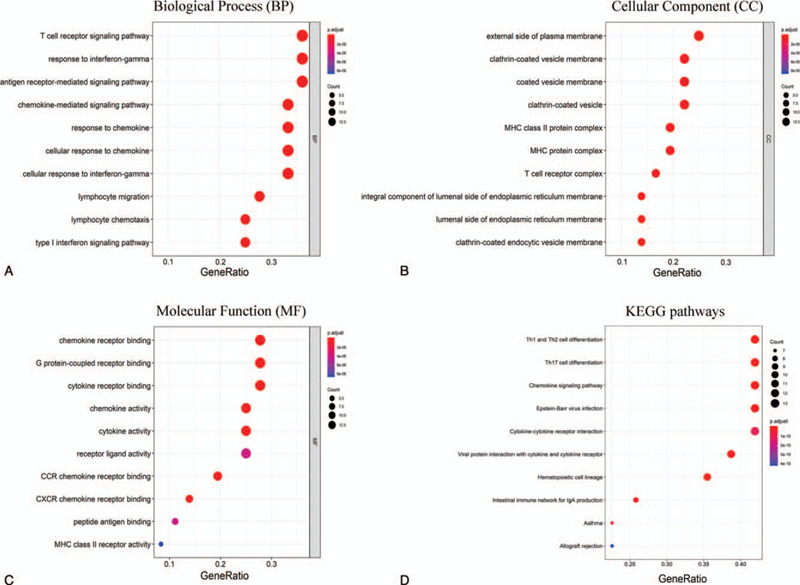
Top 10 terms of the GO and KEGG pathway. (A) Bubble diagram of the biological process sub ontologies. (B) Bubble diagram of the cell component sub ontologies. (C) Bubble diagram of the molecular function sub ontologies. (D) Bubble diagram of the top 10 terms of the KEGG pathway. GO = gene ontology, KEGG = Kyoto Encyclopedia of Genes and Genomes.

Potential biological functions of 36 upregulated DEGs in the high immune score group were investigated using the R package clusterProfiler. The GO enrichment analyses consisted of 3 subontologies: BP, CC, and MF. For the BP category, 36 DEGs were significantly enriched in the T cell receptor signaling pathway, response to interferon-gamma, and antigen receptor-mediated signaling pathway (Fig. 4A). The 36 DEGs in the CC category primarily clustered in the external side of the plasma membrane (Fig. 4B). Regarding the MF category, DEGs were mainly involved in chemokine receptor binding, G protein-coupled receptor binding, and cytokine receptor binding (Fig. 4C). Moreover, the result of the KEGG pathway analysis suggested that 36 DEGs were significantly related to immune response, such as Th1 and Th2 cell differentiation, Th17 cell differentiation, chemokine signaling pathway, etc. (Fig. 4D).

### 3.5 IRF1 expression associated with metastasis

**Figure 5:**
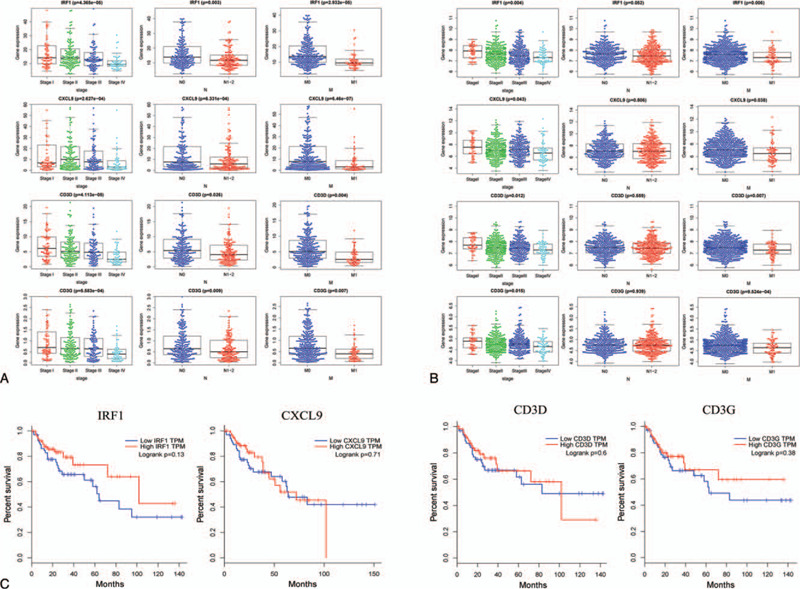
Verification of the association between 4 DEGs and metastasis. (A) Boxplots of IRF1, CXCL9, CD3D, and CD3G expression in different groups according to tumor stage, lymphatic metastasis, and distant metastasis based on TCGA database. (B) Boxplots of IRF1, CXCL9, CD3D, and CD3G expression based on the GEO database. CD3D, CD3G, CXCL9, and IRF1 showed significant difference in the tumor stage and distant metastasis groups. IRF1 expression may be associated with lymph node metastasis (*P* = .052) (C) Kaplan–Meier survival curve of IRF1, CXCL9, CD3D, and CD3G based on the GEPIA. The group with high levels of IRF1 expression showed a longer overall survival than that with low IRF1 expression levels; however, there was no statistically significant difference found in the CXCL9, CD3D, and CD3G groups. CXCL9 = C-X-C motif chemokine ligand 9, DEG = differentially-expressed gene, GEO = Gene Expression Omnibus, IRF1 = interferon regulatory factor 1.

Correlation analysis was performed between 36 upregulated DEGs and clinical characteristics, including tumor stage, lymph node metastasis, and distant metastasis using the R package beeswarm. A total of 12 DEGs showed significant correlation in TCGA database (Table 2). Subsequently, we extracted the gene expression of 12 DEGs from the GSE40967 dataset to verify the reproducibility of its correlation with clinical characteristics. As shown in Table 3, the expression of CD3D, CD3G, CXCL9, and IRF1 were significantly associated with tumor stage and distant metastasis. The expression of IRF1 may be associated with lymph node metastasis (*P* = .052); there were no significant correlations found between the expression of CD3D, CD3G, and CXCL9 and lymph node metastasis in the GSE40967 dataset (Fig. 5A, B). Further Kaplan–Meier survival curves were constructed to investigate the prognostic value of these 4 genes based on GEPIA. The survival analysis indicated that patients with higher levels of IRF1 expression had a longer overall survival than those with lower levels. There were no statistically significant differences found in the CXCL9, CD3D, and CD3G groups (Fig. 5C).

**Table 2:**
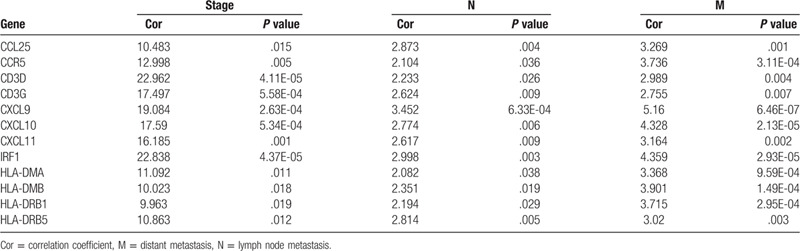
Based on TCGA, 12 DEGs significantly associated with metastasis.

**Table 3:**
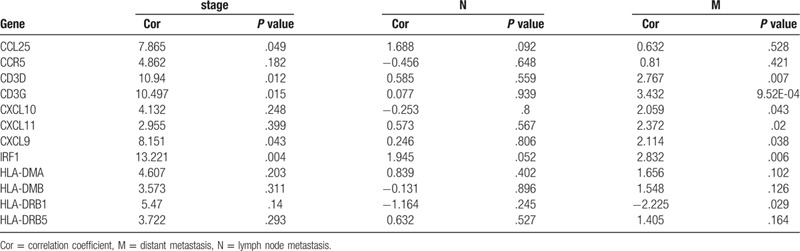
Correlation between 12 DEGs and clinical characteristics in GSE40967 dataset.

### 3.6 Immune infiltration analysis of IRF1

The association between IRF1 expression and immune infiltrates was investigated in the TIMER database. The results demonstrated that IRF1 expression was significantly positively correlated with the infiltrating degree of B cells (*r* = .111, *P* = 2.56e-02), CD8+ T cells (*r* = .229, *P* = 3.08e-06), neutrophils (*r* = .501, *P* = 6.52e-27), and dendritic cells (DC) (*r* = .406, *P* = 2.25e-17). There was a negative correlation between IRF1 expression and tumor purity (*r* = .258, *P* = 1.24e-07). In addition, there were no significant correlations between IRF1 expression and infiltrating degree of CD4+ T cells and macrophages.

**Figure 6:**
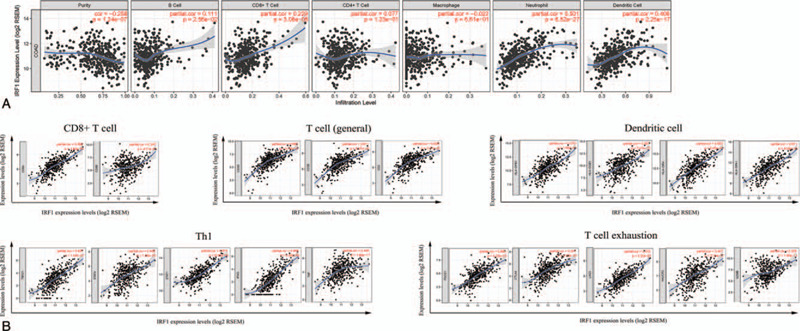
Correlation of IRF1 expression with immune cells in COAD. (A) Scatter plots of the gene module. IRF1 expression levels showing moderate positive correlations with the infiltration levels of neutrophils and dendritic cells. (B) Scatter plots of the correlation module. IRF1 expression showing strong positive correlations with some gene markers of CD8+ T, T (general), Th1 cells, dendritic cells, and T cell exhaustion. COAD = colon adenocarcinoma, IRF1 = interferon regulatory factor 1, Th1 = T helper 1.

In the correlation module, we focused on exploring the correlations between IRF1 and immune marker gene sets of diverse immune infiltrating cells. Following adjustment by purity, we discovered that IRF1 expression was strongly correlated with CD8A of CD8+ T cells, CD3D, CD3E, CD2 of T cell (general), T-box transcription factor 21 (TBX21), signal transducer and activator of transcription 1 (STAT1), and interferon gamma (IFNG) of Th1. The high infiltration degree of CD8+ T cells, T cell (general), and Th1 generally indicated that these are anti-tumor factors in COAD (Table 4). In addition, the correlations between IRF1 expression and HLA-DRA, HLA-DPA1 of DCs, and PD-1, lymphocyte activating gene 3 (LAG3) of T cell exhaustion were strong. PD-1 and LAGs play important roles in the progression of tumors. Therefore, the results of the correlation analysis further confirmed the crucial role of IRF1 in immune infiltration (Table 4). The scatter plots show each pair of IRF1-marker genes with a strong correlation (Fig. 6).

**Table 4:**
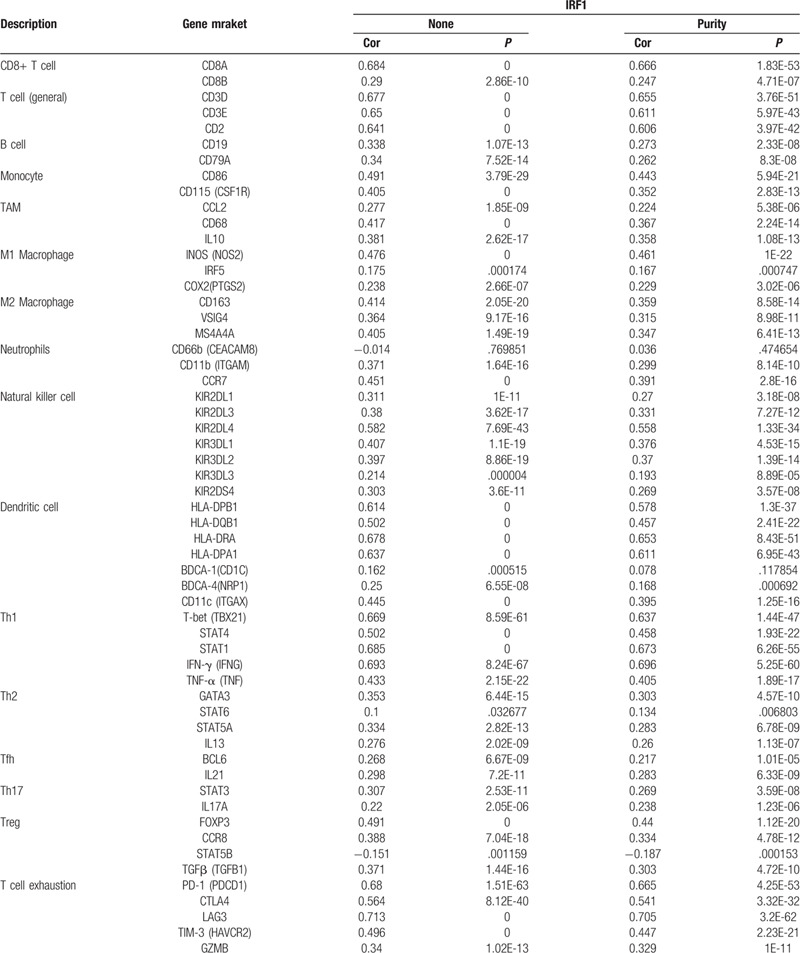
Correlation analysis between IRF1 expression and marker gene sets of immune infiltrating cells in TIMER database.

## 4 Discussion and conclusions

Metastasis of COAD to external organs is responsible for the death of most patients with this disease; hence, metastasis is linked to poor prognosis for patients.^[24]^ As new technologies are applied to investigate metastasis, the underlying mechanisms are becoming clear. Tumor metastasis is not always caused by primary tumor cells alone; the microenvironment surrounding primary tumor cells plays a critical role.^[25–27]^ Experimental evidence suggested that the TME of progressive tumors exhibits different characteristics from that of early tumors in COAD. The activated transforming growth factor-β (TGF-β) signaling in TME drives an important mechanism of immune evasion, which accelerates T cell exhaustion and inhibits acquisition of the Th1 effector phenotype.^[28]^ Blockade of TGF-β signaling unleashes a potent response of cytotoxic T cells to prevent tumor cell metastasis.^[29]^ Increased growth differentiation factor 15 (GDF15) in the TME represents a factor of tumor cell invasion and metastasis.^[30]^ In this study, we aimed to identify MRGs by analyzing the TME characteristics of different stages in COAD.

According to the distribution of the immune score in 3 groups, metastatic COAD had a lower immune score than primary COAD. The distribution characteristic of the immune score indicated that there was a powerful immune evasion in the TME of metastatic COAD; however, the factors involved in this process remain unclear. Based on the MCODE module analysis in the PPI network, 36 upregulated DEGs in the high immune score group were identified as the hub genes which drive immune evasion in metastatic COAD. The GO term and KEGG pathway enrichment analyses revealed that these 36 DEGs were mainly concerned with the process of immunity and inflammatory responses, especially for T cell-mediated immunity, such as the T cell receptor signaling pathway (BP) and Th1 and Th2 cell differentiation (KEGG). Correlation analysis using TCGA and GEO databases suggested that 4 DEGs (IRF1 CD3D, CD3G, and CXCL9) correlated with tumor stage and distant metastasis. The Kaplan–Meier survival curves of these 4 DEGs in the GEPIA showed that upregulation of IRF1 was a protective factor for the prognosis of patients with COAD. The aforementioned results indicated that the expression of IRF1 may serve as an important function in regulating immune evasion.

Indeed, as a member of the interferon regulatory factor family, IRF1 is associated with immune cell infiltration in COAD. Studies have reported that the deletion of IRF1 altered the type and function of immune cells in chronic inflammation of the colon, which increase the susceptibility of colitis-associated colon cancerer.^[31]^ The activated IRF1 recruited monocytes and macrophages to enhance the anti-tumor immune response by inducing interferon-β autocrine signaling.^[32]^ However, the mechanisms of IRF1 in immune cell infiltration are not well understood. Further correlation analysis using the TIMER database showed that IRF1 was positively correlated with CD8+ T cells, T cells (general), and Th1 cells. A previous study confirmed that patients with melanoma who were refractory to adoptive T cell therapy had reduced IRF1 expression levels.^[33]^ IRF1 influences the antitumor efficacy of cyclophosphamide by regulating the amplification of Th1.^[34]^ In contrast to Th1, Th17 cells, which were weakly correlated with IRF1, exerted opposite effects on the survival of patients with colorectal cancer.^[35,36]^ The aforementioned findings were consistent with the results of the correlation analysis, which suggested that IRF1 served as an antitumor factor in COAD. Although there was no direct evidence that IRF1 was correlated with HLA-DRA or HLA-DPA1 of DCs, the correlation between IRF1 and major histocompatibility complex class I (MHC-I) molecules has been reported. In aggressive neuroblastoma, IRF1 and nuclear factor-κB rescued the immune escape phenotype by restoring the pathway of MHC class I-restricted tumor antigen processing and presentation to cytotoxic T lymphocytes.^[37]^ Certainly, with the improved understanding of tumor immunology, increasing attention has been focused on the gene markers of the MHC-II restricted neoantigen.^[38]^ These correlation between IRF1 and HLA-DRA and HLA-DPA1 indicated a differential perspective of tumor immunology.

The correlation analysis also yielded another unexpected result. IRF1 showed a strong correlation with PD-1 and LAG3 of T cell exhaustion, which are both tumor-promoting factors in the TME.^[39–41]^ These results indicated a differential function of IRF1 in COAD. Studies have revealed that PD-1 led to T cell apoptosis, impairing protective inflammatory response and promoting tumor immune evasion.^[42]^ Nevertheless, preliminary clinical studies of PD-1/PD-L1 checkpoint inhibitors in colon cancer showed that the effectiveness of these treatments was extremely limited except for the microsatellite instability subtype.^[18,27]^ IRF1 is a transcription factor of the interferon receptor signaling pathways, which primarily regulate the expression of PD-L1 (a ligand of PD-1).^[43]^ A recent experiment reported that IRF1-deficient tumors exhibited enhanced cytotoxicity of CD8+ T cells through downregulation of PD-L1 expression.^[44]^ The aforementioned results imply the presence of a mechanism to explain the limited efficacy of PD-1/PD-L1 checkpoint inhibitors. Further investigation is warranted to confirm this conjecture.

In conclusion, inhibition of IRF1 expression is correlated with metastasis, which is caused by immune evasion in advanced COAD. A low degree of T cell (including CD8+ T cells and Th1 cells) infiltration caused by IRF1 deficiency is mainly responsible for the immune evasion. This result increases our understanding of the TME and may provide a potential therapeutic target for immunotherapy in colon cancer.

## Acknowledgments

The authors are thankful to all participants in this study.

## Author contributions

**Conceptualization:** Lina Meng.

**Data curation:** Yaojian Shao.

**Formal analysis:** Yaojian Shao.

**Funding acquisition:** Yaojian Shao.

**Methodology:** Lina Meng.

**Investigation:** Yaojian Shao.

**Software:** Yaojian Shao

**Supervision:** Xiong-peng Wen, Meng-die Shen.

**Validation:** Lina Meng.

**Writing – original draft:** Liting Ye, Jun-jie Ni, Shen-yu Wei.

**Writing – review & editing:** Lina Meng.
